# Platinum Accumulation and Cancer-Related Fatigue, Correlation With IL-8, TNF-α and Hemocytes

**DOI:** 10.3389/fphar.2021.658792

**Published:** 2021-09-07

**Authors:** Yuling Zhang, Xiaoting Huang, Shanna Feng, Chen Chen, Dainian Guo, Ling Fang

**Affiliations:** ^1^ Department of Pharmacy, Shantou University Medical College, Shantou, China; ^2^ Pharmacy Intravenous Admixture Service, Cancer Hospital of Shantou University Medical College, Shantou, China; ^3^ Good Clinical Practice, Cancer Hospital of Shantou University Medical College, Shantou, China

**Keywords:** platinum, accumulation, cancer-related fatigue, cytokine, hemocytes

## Abstract

Platinum-based chemotherapy drugs cause platinum accumulation and result in cancer-related fatigue (CRF), which is related to immune response through still ambiguous mechanisms. We aimed to explore the correlation between platinum and CRF from the perspective of platinum accumulation. After allowing for complete metabolism of the administered platinum drugs, we collected blood samples from 135 patients who had at least two platinum chemotherapy rounds, correlated the platinum concentration (C-Pt), pro-inflammatory cytokines IL-8 and TNF-α, hematological index with therapeutic effect, adverse reactions and fatigue. The median platinum concentration was higher in patients treated with cisplatin than oxaliplatin (424.0 vs 211.3 μg/L), and the occurrence of fatigue was 64.4% in all subjects. Separately, the incidence and degree of fatigue were 74.1% and 9.5 in the patients with higher platinum concentration compared to 57.1% and 2.0 in the lower group. C-Pt, IL-8 and TNF-α were positively correlated with the degree of CRF, while erythrocyte count and hemoglobin were negatively correlated with the degree of CRF. Mediating effect analysis showed that increased IL-8 concentration mediated 57.4%, while decreased erythrocyte count mediated 24.1% of the C-Pt effect on CRF. Platinum accumulation may involve increasing IL-8, cause inflammation or aggravate anemia, which in combination lead to CRF.

## Introduction

Platinum-based drugs are widely used in basic, combination therapy and even radiotherapy, and recognized as one of the most effective drugs for clinical treatment of malignant tumors (Gao et al., 2016; Li et al., 2019) but they have been reported to have immune toxicity-related adverse reactions (Yamauchi et al., 2015; Chopra et al., 2016), including allergy, pruritus, diarrhea, nausea, and emesis, which may be due to the effects of the immune system by killing cancer cells (Manohar and Leung, 2018). Cancer-related fatigue (CRF) has also been reported to result from chemotherapy with platinum drugs (Izgu et al., 2019; Modest et al., 2019). CRF can occur throughout the entire course of the disease, including before, during, and after treatment through the end of life (Bower, 2014). Early CRF may be caused by a tumor or cancer cells releasing cytokines or causing oxidative stress. During treatment, CRF may be related to surgery, chemotherapeutic drugs, inflammation or radiation therapy. Fatigue in the later stages may also be related to changes in the immune system, such as altered lymphocyte subsets and anemia (Bower, 2014; Koornstra et al., 2014; Aapro et al., 2017). To date, the mechanisms of platinum-based drugs leading to CRF remain unclear.

During clinical therapy, we found that some patients with stable disease, who had been treated with platinum drugs, still had obvious fatigue after the time required for the theoretical complete metabolism of the original drug, suggesting that CRF may related to platinum accumulation. Although there is no direct evidence on the mechanism of the relationship between platinum accumulation and CRF, a prior study has shown dysregulated mitochondrial and energy homeostasis caused by platinum accumulation (McQuade et al., 2018). Once the structure and function of mitochondria are destroyed, the energy supply of cells will be reduced, leading to various uncomfortable symptoms, such as fatigue (Yang et al., 2019). Moreover, platinum can competitively replace copper (Cu^2+^), reduce the transport of Cu^2+^, and lead to a decrease in the mitochondrial Cu^2+^ pool, which is critical for complex IV and COX17 function, as well as oxidative phosphorylation, thereby decreasing ATP production (Lutsenko et al., 2007; Howell et al., 2010). For other heavy metals, several have also been reported to cause immune system damage, oxidative stress and anemia by interfering with interleukins (ILs) and metallothionein, or by competitive inhibition of iron ions (Biswas et al., 2008; Mishra, 2009; Sharma et al., 2010; Jorissen et al., 2013; Hsieh et al., 2017; Zhang et al., 2020a). Furthermore, some pro-inflammatory cytokines, such as IL8 and TNF-α, produced by macrophages, eosinophils, and B cells, have a role in initiation of the acute immune response (Oliveira Miranda et al., 2014) and correlate with fatigue (Bower, 2014; Wu et al., 2019). An association between elemental platinum and cytokines has not been reported, but cisplatin has been shown to influence TNF-α level (Chtourou et al., 2016). In addition, fatigue of some non-cancer patients has been related to an allergy to inorganic mercury and nickel, and such metal driven inflammation may affect the hypothalamus-pituitary-adrenal axis (HPA axis) to indirectly trigger psychosomatic symptoms characterized by chronic fatigue syndrome, fibromyalgia and other diseases of unknown etiology (Sterzl et al., 1999). The fatigue of workers exposed to mercury is also related to the level of mercury (Ekinci et al., 2014). Lead may affect glycolysis and the pentose phosphate pathway, thus interfering with ATP synthesis and resulting in fatigue (Owsianowska et al., 2020). Associations between platinum, immune related cytokines and CRF have not been reported. As a divalent heavy metal, does the accumulation of platinum induced by chemotherapeutic drugs cause reactions similar to those of other heavy metals?

Given the above, we attempted to assess the relationships between platinum accumulation and immune system-related indices, including cytokines and hemocytes, to explore the mechanism of CRF on the perspective of platinum, a heavy metal induced by chemotherapy.

## Materials and Methods

### Sample Collection

We randomly recruited patients who had undergone platinum drug chemotherapy, in the Cancer Hospital of Shantou University Medical College, after diagnosis by a doctor according to the NCCN (National Comprehensive Cancer Network) guidelines. For some patients who had undergone surgery, a postoperative recovery period and assessment by a doctor were implemented before chemotherapy. All patients were eligible for enrollment, and exclusion conditions before medication are shown in Table 1.

**TABLE 1:** Recruitment criteria.

Enrollment criteria	Exclusion criteria
Estimated lifetime >6 months	Pregnant or lactating
Required platinum drug chemotherapy after diagnosis	Metallurgical industry employee; Metastatic tumor
Hematological index within the normal range	Prior medication involving platinum or other chemotherapy; Therapeutic effect is progressive
Provided informed consent to this research	Accompanied by inflammation

As we have reported before, oxaliplatin and cisplatin have a half-life of 46 and 72 h, respectively, so the time required for complete metabolism should be 10.5 and 16.5 days or more, respectively, based on the theory of 5.5 times the half-life (Zhang et al., 2020b). Hence, the chemotherapy cycle was set for more than 3 weeks according to drug elimination and the clinical requirements of the NCCN. We collected samples on the day before the next round of chemotherapy, to ensure ample time for metabolism. Ultimately, 135 cases were obtained, from January to December in 2018, after eliminating invalid samples, 77 cases for oxaliplatin and 58 cases for cisplatin. A total of 4 ml of elbow venous blood was obtained each patient, and 1 ml was analyzed immediately for hematological index, while 3 ml was centrifuged (1,200 g, 3 min) to obtain serum for platinum measurement.

### Ethics Approval

All subjects gave their informed consent for inclusion before participating in the study. The study was conducted in accordance with the Declaration of Helsinki, and the protocol was approved by the Human Ethics Committee of the Cancer Hospital of Shantou University Medical College, China (2015030907).

### Measurement of Serum Platinum

Methods and parameters for the equipment were implemented according to our prior article (Zhang et al., 2020b). A total of 100 μL serum sample was used for platinum element analysis. Nitric acid (65%, guarantee) was added to the serum sample and the solution was analyzed by graphite furnace atomic absorption spectrophotometry (Jena ZEEnit 650, Germany). Parameters for the AAS-650 were a 265.9 nm wavelength, 0.2 nm slit width, and 8 mA Pt-lamp current. The limit of detection (LOD) was 31.16 μg/L and accuracy of this method was verified by recoveries on 94.77% from spiked serum samples.

### Hematological Index Analysis

Hematological indices were measured with an automatic blood analyzer (Beckman-LH780, United States) immediately after sample collection by impedance measurement and cyanated methemoglobin colorimetric procedures.

### Measurement of IL-8 and TNF-α

Cytokines were detected in serum samples with the Cytometric Bead Array (CBA) for Human IL-8 and TNF-α (Enhanced Sensitivity Flex Set, BD™, United States), using a BD Accuri C6 flow cytometer.

### Evaluation of Adverse Reactions, Chemotherapeutic Effect and Fatigue

Adverse reactions and chemotherapeutic effect were assessed and recorded at the sample collection time by a clinical doctor, based on The Common Terminology Criteria for Adverse Events (CTCAE 5.0) and Response Evaluation Criteria in Solid Tumors (RECIST 1.0). Adverse reactions, including diarrhea, nausea, emesis and allergy, were classified into five grades (from 0-no symptoms to 4-severe life-threatening), while the chemotherapeutic effect was assessed and classified by four grades (1-progressive disease, PD; 2-stable disease, SD; 3-partial remission, PR; 4-complete remission, CR).

The Chinese version of the Brief Fatigue Inventory Scale (BFI-C) was used to assess the CRF of each patient on the same day as the sample collection. The BFI-C is a 9-item questionnaire. For each item, 0 indicates no fatigue and 10 indicates the most severe fatigue. The sum of each item score was defined as the total score, the higher the score, the more severe the fatigue.

### Statistical Analysis

Nonparametric analysis was performed, using the Kolmogorov-Smirnov test, for frequency distribution. Data with skewed distributions were represented by the median and 25th-75th percentile, and skewed data were compared by non-parametric testing (Mann Whitney U test). The comparison of rates was performed by the chi-square test, and correlation analysis was performed by Spearman rank correlation analysis. A natural logarithm transformation was used to construct approximate normal distributions. The concentration-response relationship was analyzed by multiple linear regression. All analyses were performed with SPSS 22.0 (IBM Corporation, United States) and GraphPad Prism 7.0 (GraphPad, CA) software. A *p* < 0.05 was considered as statistically significant in all analyses.

## Results

### General Characteristics of the Study Population

We obtained 135 cases of platinum-treated patients, 77 for oxaliplatin and 58 for cisplatin. General characteristics of the patients are shown in Table 2. No significant differences were observed for gender, age, height, weight and BMI between the two groups (*p* > 0.05). The original levels of platinum in the two groups were equivalent, based on calculations of drug structure and dose.

**TABLE 2:** General characteristics of the study population.

General characteristics	Oxaliplatin (n = 77)	Cisplatin (n = 58)	Statistics	*p-*value
Gender (males/females)	55/22	35/23	χ^2^ = 1.829	0.176
Age (mean ± SD, years)	59.68 ± 10.64	56.02 ± 10.43	*t* = 1.994	0.048
Height (mean ± SD, cm)	164.71 ± 5.93	164.36 ± 6.45	*t* = 0.329	0.743
Weight (mean ± SD, kg)	55.53 ± 6.62	55.00 ± 7.09	*t* = 0.443	0.658
BMI (mean ± SD, kg/m^2^)	20.41 ± 1.52	20.27 ± 1.51	*t* = 0.483	0.630
Surgery [n (%)]			χ^2^ = 22.718	0.000
1 = No	34 (44.2)	49 (84.5)		
2 = Yes	43 (55.8)	9 (15.5)		
Dose (mg/m^2^)	85	65	—	—

### Serum Platinum, Hemocytes and Cytokine Levels

**FIGURE 1:**
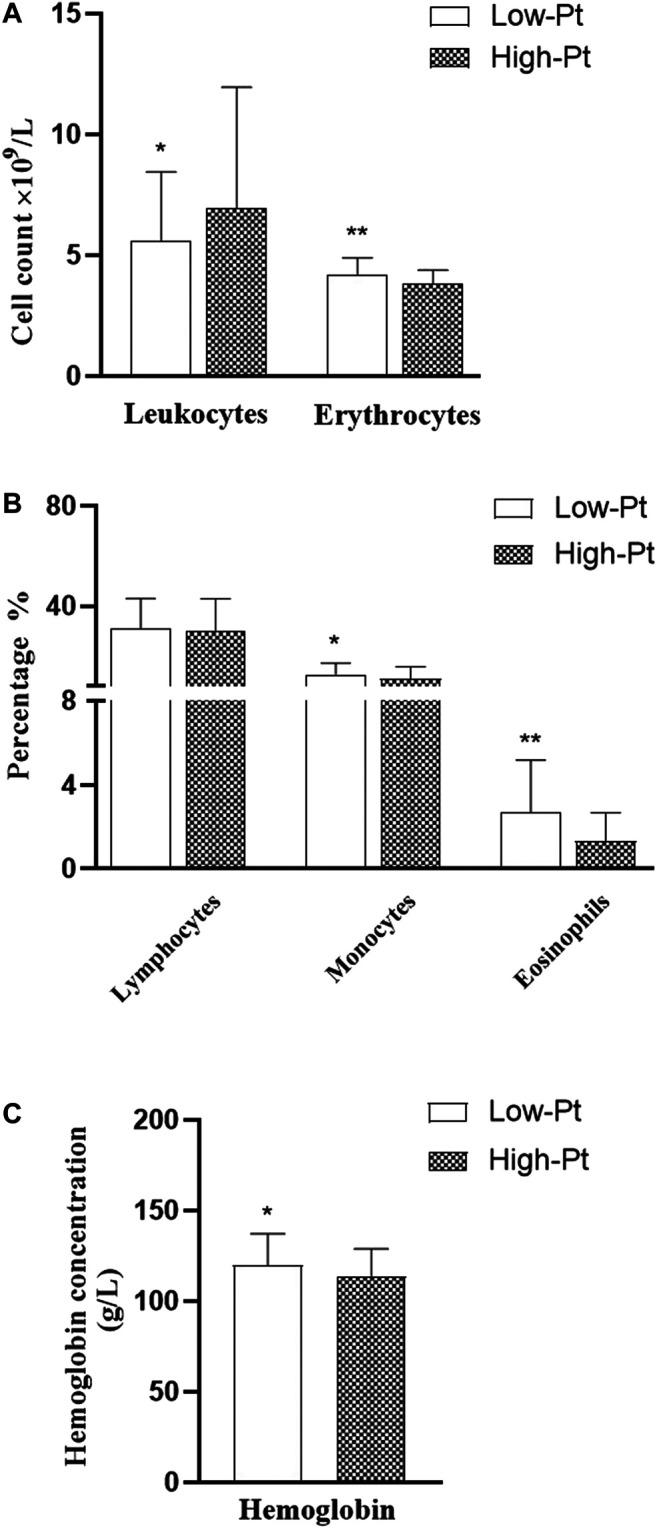
Comparison of hemocytes in the low- and high-platinum groups. Low-Pt group, n = 77, High-Pt group, n = 58. a: leukocyte and erythrocyte counts b: percentage of lymphocytes, monocytes and eosinophils c: level of hemoglobin. **: *p* < 0.01; *:*p* < 0.05.

**FIGURE 2:**
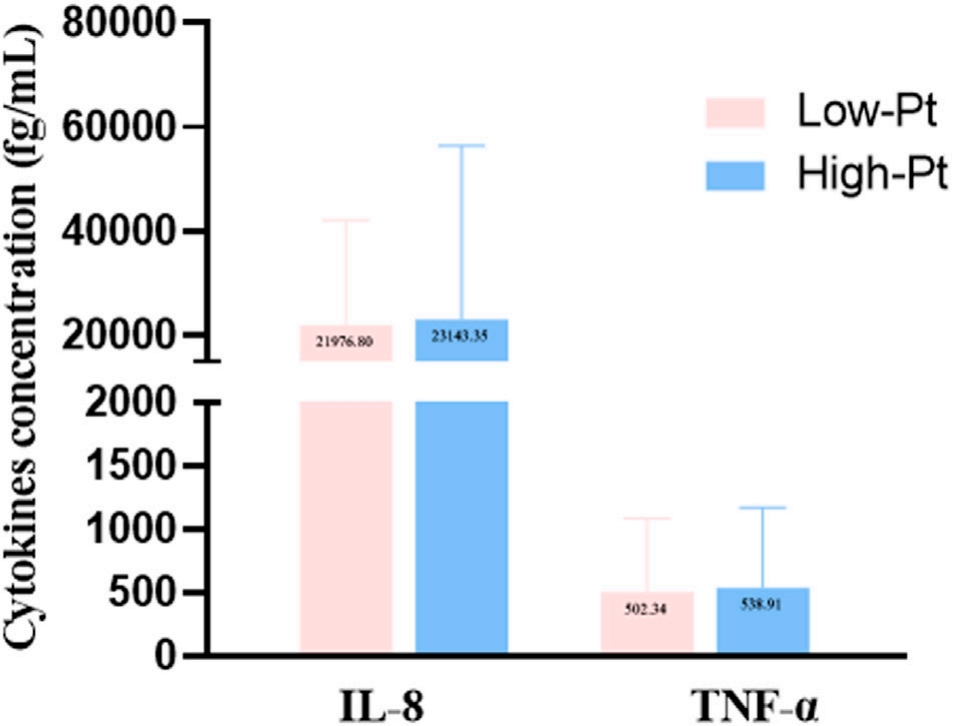
Cytokine levels in the low- and high-platinum groups. Low-Pt group, n = 77, High-Pt group, n = 58.

The serum platinum concentration (C-Pt) showed a non-normal distribution. The median platinum concentration was 211.3 (160.3, 283.6) μg/L in the oxaliplatin group and 424.0 (343.7, 537.4) μg/L in the cisplatin group (Mann-Whitney Test, *Z* = -7.790, *p* = 0.000). We divided all cases into low- and high-platinum groups by the mean of the natural logarithm of platinum concentration. Hematological indices of leukocyte and erythrocyte counts, percentages of lymphocytes, monocytes and eosinophils, and levels of hemoglobin (Figure 1), levels of IL-8 and TNF-α (Figure 2) were compared between the two groups. The leukocyte count was lower (5.59 vs 6.94 ×10^9^/L, *p* < 0.05), but erythrocyte count (4.20 vs 3.82 ×10^9^/L, *p* < 0.05) and hemoglobin level (119.92 vs 113.74 g/L, *p* < 0.05) were higher in the low-platinum group compared to the high-platinum group. The percentages of monocytes (12.73 vs 10.99%, *p* < 0.05) and eosinophils (2.70 vs 1.32%, *p* < 0.05) were also higher in the low-platinum group compared to the high platinum group. No significance was found in lymphocyte percentage, IL-8 and TNF-α between the two groups (31.16 vs 29.93%, 21976.80 vs 23143.35 fg/ml, 502.34 vs 538.91 fg/ml, respectively, *p* > 0.05).

### Degree of Cancer-Related Fatigue

**FIGURE 3:**
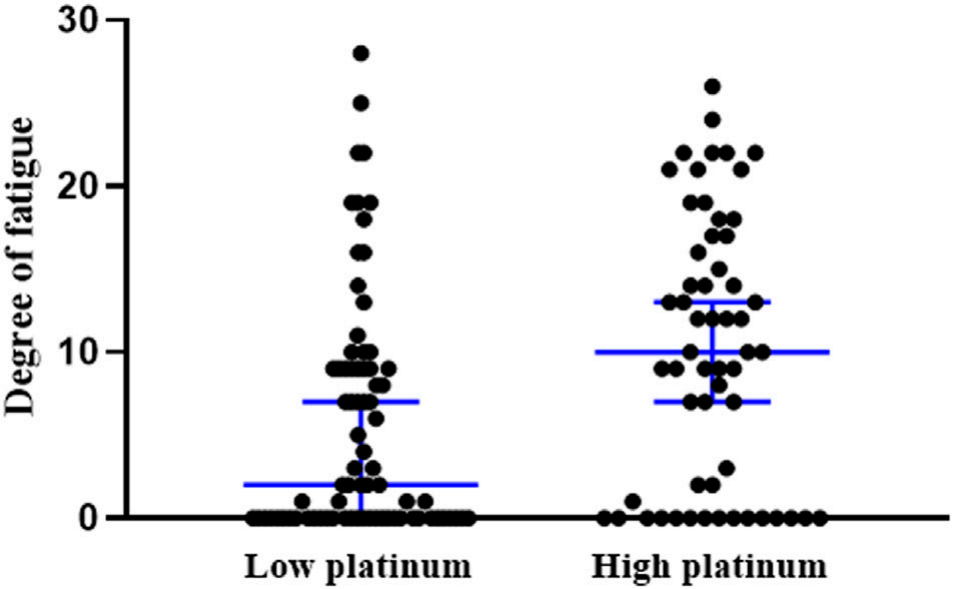
Scatter plot on the degree of fatigue in low- and high-platinum groups. **:p < 0.01.

The occurrence of CRF was 64.4% in all subjects. Comparison of the occurrence and the degree of CRF indicated a higher incidence of occurrence in the high platinum group than in the low-group (74.1 vs 57.1%, χ^2^ = 4.170, *p* = 0.041), and also greater fatigue severity in the high platinum group (*p* = 0.002, Figure 3).

### Factors Impacting the Degree of Cancer-Related Fatigue

We used Spearman correlation analysis to explore the factors impacting fatigue, including surgery, degree of adverse reactions (diarrhea, nausea, emesis and allergy), therapeutic effect and C-Pt. Results showed that the degree of CRF was positively associated with the degree of adverse reactions and C-Pt, but negatively associated with therapeutic effect (*p* < 0.05, Table 3). Analysis of hemocytes, IL-8 and TNF-α showed that the concentrations of IL-8 and TNF-α were positively, but erythrocyte and hemoglobin were negatively correlated with the degree of CRF (*p* < 0.05, Table 3).

**TABLE 3:** Spearman correlation analysis on CRF and impact factors.

Factors	*r*_s_	*p*-value
Surgery	−0.085	0.325
Adverse reaction	0.224*	0.009
Therapeutic effect	−0.230**	0.007
C-Pt	0.304**	0.000
IL-8	0.305*	0.030
TNF-α	0.465*	0.017
Erythrocytes	−0.250**	0.000
Hemoglobin	−0.199*	0.021
Leukocytes	0.085	0.330
Platelets	0.037	0.668
Lymphocyte %	−0.012	0.889
Monocyte %	−0.048	0.579
Eosinophil %	−0.079	0.364

### Correlations Between C-Pt, Hematopoiesis, Cytokines and Cancer-Related Fatigue

To explore the associations between C-Pt and the related factors of hemocytes and cytokines, linear regression models were used, with C-Pt as the independent variable. After adjustment for patient gender, age, BMI and cancer types, we found an increasing trend for IL-8 levels but a decreasing trend for erythrocyte counts with the increase of C-Pt (Table 4, *p* < 0.05).

**TABLE 4:** Correlations between cytokines, hemocytes and C-Pt.

	C-Pt		
	B (95% CI)	β	*p*-value
Erythrocytes	−0.001 (−0.001, 0.000)	−0.180	0.034
Hemoglobin	−0.006 (−0.020, 0.009)	−0.066	0.447
IL-8	63.870 (26.710, 101.030)	0.473	0.001
TNF-α	−0.323 (−1.595, 0.949)	−0.125	0.603

C-Pt: the concentration of platinum.

Data analysis with adjustment of age, gender, BMI and cancer types.

**FIGURE 4:**
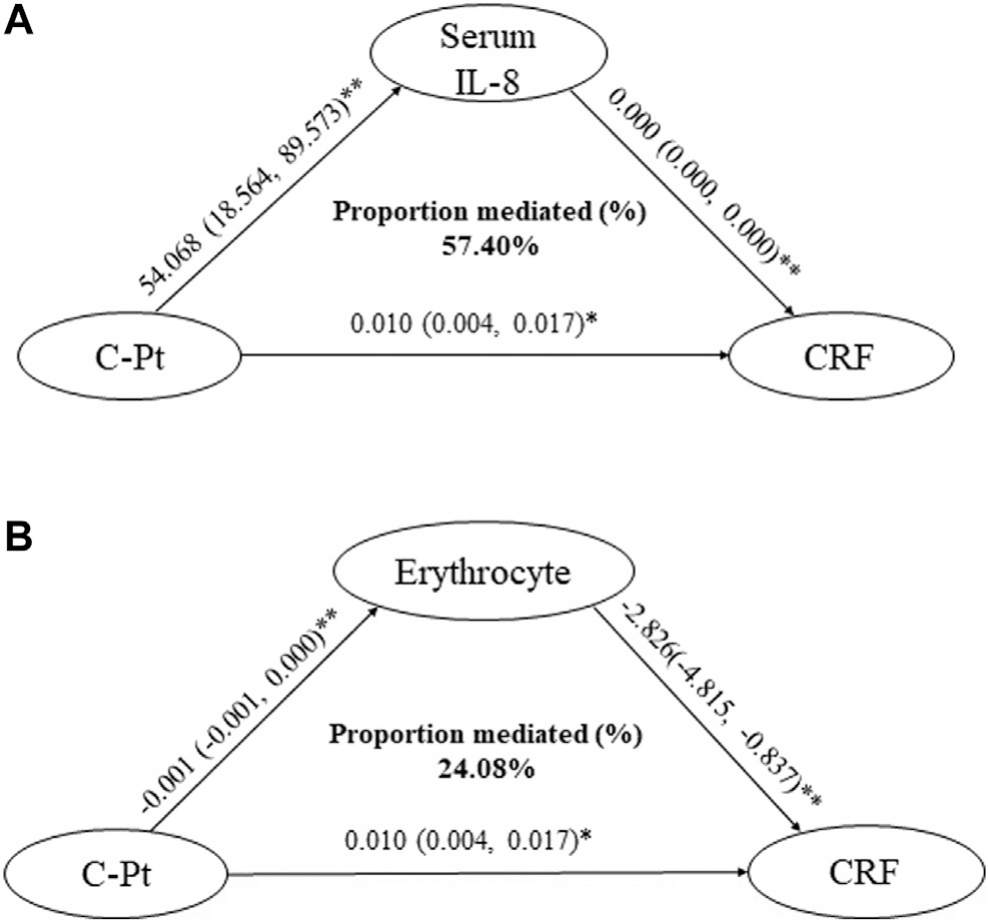
Mediation effect assessments of IL-8 and erythrocytes on the association of C-Pt with CRF. a: mediation effect of serum IL-8 level on the association of C-Pt with CRF, *p* < 0.05. b: mediation effect of erythrocyte counts in whole blood on the association of C-Pt with CRF, *p* < 0.05. *: *p* < 0.05; **: *p* < 0.01.

After the regression analysis with C-Pt as the independent variable and the degree of CRF as the dependent variable, each 1 μg/L increase in C-Pt was associated with a 0.010-fold increase of the degree of CRF (95% CI: 0.004, 0.017). Mediating effect analysis showed increased IL-8 concentration mediated 57.40% of the C-Pt effect on the degree of CRF, whereas decreased erythrocyte count mediated 24.08% of the C-Pt effect on the degree of CRF (Figure 4A, 4B. *p* < 0.05).

## Discussion

### Comparison of Platinum in Different Drugs

In this study, we found a high level of platinum accumulation in cancer patients, with a higher concentration in the cisplatin group than in the oxaliplatin group, even after a time when complete metabolism of the administered drugs should have occurred. Differences in platinum level may be due to drug structure, with different rates of metabolism (Boisdron-Celle et al., 2001; Sprowl et al., 2012; Wang et al., 2017). This is similar to other research showing the presence of platinum 20 years after therapy (Linde et al., 2017), and the elimination time for platinum being more than 25 times that for the original platinum-based drug (Qin et al., 2019).

### Comparison of Cancer-Related Fatigue in Different Platinum Level Groups

Researchers have reported an 88% incidence of fatigue before chemotherapy (Sha et al., 2015), 17–53% during chemotherapy and very serious fatigue in some patients with locally advanced colorectal cancer and esophageal cancer (Koornstra et al., 2014). Persistent fatigue can last for 5–10 years in 20–30% of cancer survivors (Bower et al., 2006). Therefore, CRF does not only occur during chemotherapy, but also may last for a long time after treatment following complete metabolism of the original drugs. We found 64.4% of patients experience fatigue following complete platinum drug metabolism. The incidence is significantly higher in the high platinum group, which suggests a correlation between CRF and platinum accumulation.

### Factors Impacting Cancer-Related Fatigue

In this study, CRF was negatively correlated with the chemotherapeutic effect, with the better the effect, the lower the incidence of fatigue. Obviously, better treatment effect could improve mood and sleep, while relieving the stress and cancer pain of the patients, all of which could alleviate fatigue. On the other hand, CRF was positively correlated with adverse reactions related to the immune system, with more severe adverse reactions being associated with a higher incidence of fatigue. Adverse reactions, such as nausea, vomiting and diarrhea, may disturb the normal functions of the body. For example, excessive diarrhea can cause lower limb weakness, and frequent vomiting can easily aggravate the gastrointestinal discomfort of patients and make them tired. This suggests a correlation between immune function and CRF in this study.

Erythrocyte count and hemoglobin level showed negative correlations with the degree of CRF. Erythrocyte and hemoglobin levels directly determine oxygen carrying capacity. Low levels of both can lead to anemia, resulting in an insufficient oxygen supply, which can make people feel tired and is closely related to fatigue (Aapro et al., 2017). On the contrary, increasing hemoglobin can improve the functional status of patients (Jakel, 2002). Moreover, erythrocytes also play a role in immune regulation by taking part in cell adhesion, migration, and cell-cell interactions (Huo et al., 2019). The correlations between erythrocyte count, hemoglobin level and degree of CRF may suggest mechanisms on CRF related to anemia and immunoregulation.

Positive correlations between IL-8, TNF-α and CRF found in the present study are in line with previous research. As one of pro-inflammatory cytokines, IL-8 is associated with fatigue in breast cancer patients (Cohen et al., 2020), and increased levels of TNF-α can cause mitochondrial damage and result in fatigue (Muluye et al., 2016), Polymorphisms in TNF-α have also been reported in association with fatigue in a small longitudinal study of prostate cancer patients (Jim et al., 2012; Bower, 2014).

Some reports have pointed out that heavy metals impact the hemocyte and immune system. For example, lead negatively correlates with erythrocyte adhesion molecules (CD44 and CD58) and reduces the concentration of hemoglobin (Hsieh et al., 2017; Weinhouse et al., 2017; Huo et al., 2019). Lead exposure causes immune hypersensitivity and increases leukocyte counts in some animal studies (Xu et al., 2015), and cadmium indirectly generates ROS through its ability to substitute for iron and copper (Wu et al., 2016). From the aspect of lead on anemia, a high level of lead is associated with iron deficiency (Shah et al., 2010). Linear regression analysis in our research has shown that the accumulation of platinum had a positive effect on IL-8, but negative effect on erythrocytes. Mediating effect analysis showed the influence of platinum accumulation on IL-8 levels and erythrocyte counts leading to CRF, indicating that platinum may interfere with immune function through induction of pro-inflammatory cytokines, competitively inhibiting iron or influencing adhesion, and reducing levels of erythrocytes and hemoglobin to cause or aggravate fatigue after chemotherapy.

In this study, adverse reactions, chemotherapeutic effects, erythrocyte counts, hemoglobin level, IL-8, TNF-α and C-Pt correlated with CRF. The percentages of monocytes and eosinophils were different in low- and high-platinum group but have no correlations with CRF. We also failed to observe a relationship between leukocytes and CRF, perhaps due to the small sample size and clinical difficulty. The mechanism of CRF still requires further research, including of other cytokines and TNF receptors, which have also been reported to correlate with fatigue after chemotherapy (Collado-Hidalgo et al., 2006). Fatigue influences the quality of life and even shortens survival time of cancer patients (Aapro et al., 2017). Regarding clinical therapy and nursing, platinum detection before the next chemotherapy will be conducive to dosage adjustment, based on the accumulation of elemental platinum, quelation therapy could be considered for platinum chelation and improvement of quality life. Hemoglobin supply can also be considered as a nutritional supplement for patients after chemotherapy. This is the first time the mechanism of cancer-related fatigue, induced by platinum accumulation, has been correlated with immune-related cells and cytokines. We will further explore the mechanism of platinum from the perspective of cell pathways in the future.

## Data Availability

The raw data supporting the conclusions of this article will be made available by the authors, without undue reservation.
